# Detecting Fragmentation Extinction Thresholds for Forest Understory Plant Species in Peninsular Spain

**DOI:** 10.1371/journal.pone.0126424

**Published:** 2015-05-15

**Authors:** Marta Rueda, Juan Carlos Moreno Saiz, Ignacio Morales-Castilla, Fabio S. Albuquerque, Mila Ferrero, Miguel Á. Rodríguez

**Affiliations:** 1 Department of Ecology and Evolutionary Biology, University of California Irvine, Irvine, California, United States of America; 2 Forest Ecology & Restoration Group, Department of Life Sciences, Universidad de Alcalá, Alcalá de Henares, Madrid, Spain; 3 Department of Biology (Botany), Universidad Autónoma de Madrid, Madrid, Spain; 4 Department of Biology, McGill University, Montreal, Quebec, Canada; 5 School of Forestry, Northern Arizona University, Flagstaff, Arizona, United States of America; Universidade Federal de Goiás, BRAZIL

## Abstract

Ecological theory predicts that fragmentation aggravates the effects of habitat loss, yet empirical results show mixed evidences, which fail to support the theory instead reinforcing the primary importance of habitat loss. Fragmentation hypotheses have received much attention due to their potential implications for biodiversity conservation, however, animal studies have traditionally been their main focus. Here we assess variation in species sensitivity to forest amount and fragmentation and evaluate if fragmentation is related to extinction thresholds in forest understory herbs and ferns. Our expectation was that forest herbs would be more sensitive to fragmentation than ferns due to their lower dispersal capabilities. Using forest cover percentage and the proportion of this percentage occurring in the largest patch within UTM cells of 10-km resolution covering Peninsular Spain, we partitioned the effects of forest amount versus fragmentation and applied logistic regression to model occurrences of 16 species. For nine models showing robustness according to a set of quality criteria we subsequently defined two empirical fragmentation scenarios, minimum and maximum, and quantified species’ sensitivity to forest contraction with no fragmentation, and to fragmentation under constant forest cover. We finally assessed how the extinction threshold of each species (the habitat amount below which it cannot persist) varies under no and maximum fragmentation. Consistent with their preference for forest habitats probability occurrences of all species decreased as forest cover contracted. On average, herbs did not show significant sensitivity to fragmentation whereas ferns were favored. In line with theory, fragmentation yielded higher extinction thresholds for two species. For the remaining species, fragmentation had either positive or non-significant effects. We interpret these differences as reflecting species-specific traits and conclude that although forest amount is of primary importance for the persistence of understory plants, to neglect the impact of fragmentation for some species can lead them to local extinction.

## Introduction

Key issues concerning conservation related to the anthropogenic alteration of natural habitats include the amount of habitat needed to achieve conservation goals and the importance of habitat fragmentation [1]. Habitat fragmentation is literally the ‘breaking apart’ of habitat and is usually defined as a landscape-scale process that differs from how much habitat there is [2, 3]. However, the term habitat fragmentation has often been used loosely to illustrate habitat destruction by humans even though habitat might be removed without increasing fragmentation [4]. This has generated a misunderstanding of the impacts of habitat amount and fragmentation on biodiversity [3]. Nowadays, habitat loss is still the biggest single source of pressure on biodiversity worldwide [5], and there is controversy about the effects of habitat fragmentation *per se* (i.e. independent of habitat loss effects). Conceptually, the debate extends to distinguishing habitat loss *per se* from habitat fragmentation *per se* [2, 3], or to their interdependence, recognizing that fragmentation is usually a consequence of habitat loss [6].

Ecological theory indicates that a key consequence of fragmentation *per se* is to aggravate the effects of habitat loss [7]. Theoretical models predict that the higher the fragmentation level, the earlier the appearance in the habitat loss gradient of the extinction threshold; that is, of the minimum amount of habitat below which a population cannot persist and becomes locally extinct [1, 8, 9]. However, this so-called fragmentation threshold hypothesis [10, 11] has received tenuous support from empirical results [3], which may be associated with how habitats are defined in theoretical *vs*. empirical studies [12, 13]. The habitat concept has a clear species-specific nature, referring to the resources and conditions permitting occupancy of space by a particular species [14]. As such, habitat is introduced in most theoretical studies with the characteristics that researchers want to assign to it. Conversely, gaps of knowledge and data often preclude such a close species-habitat matching in empirical studies, which typically use broad, human-perceived habitat types (e.g. forest) to which groups of species are assigned as if the species integrating each group had identical habitat needs (e.g. forest specialists) [4, 12]. Due to these limitations, mismatches between empirical results and theoretical propositions are perhaps not surprising.

On the other hand, because broad vegetation and land use types often form the basis of decision making in conservation (see also [4]), the use of these habitat proxies in fragmentation studies may facilitate the transferability of empirical results to real conservation issues. Additionally, Didham et al. [6] argued for the existence of sets of species’ traits generating common responses to fragmentation of these broad habitat proxies in different species. If confirmed, such traits could be used as cues to pinpoint in advance which species are likely to be more sensitive to fragmentation and thus to define preventive conservation actions directed to them. This perspective has been supported by Rueda et al. [12] who studied how forest amount and fragmentation are related to the occurrence of seven forest bird specialists across Europe. They found that while all species were negatively affected by forest loss *per se*, two Phasianidae species with limited vagility and dispersal capabilities were also sensitive to fragmentation *per se*. These results emphasize the importance of dispersal as a determinant of species reactions to fragmentation [15–17] and illustrate how key species traits could be used as references to guide conservation actions [6].

In this paper we test the fragmentation threshold hypothesis by investigating the effects of forest loss and fragmentation on the occurrence of forest understory plant species (herbs and ferns) in Peninsular Spain, paying special attention to differences in dispersal. In general, these shade-tolerant plants often possess traits (e.g. low seed production, clonal reproduction, long-lived perennial, lack of structures for long-distance dispersal) that can be seen as evolutionary adaptations to relatively stable forest ecosystems where disturbances are generally infrequent and localized [18]. Consequently, they may suffer in open, fragmented habitats—e.g. due to inferior competitive abilities under increased levels of light [19, 20]. However, whereas seed dispersal distances of many forest herbs typically do not exceed a few meters (see [18] and references therein), dispersal of ferns is primarily via dust-like spores that can be dispersed by wind over very long distances [21, 22]. Accordingly, if responses to forest fragmentation were primarily determined by dispersal capabilities, we would expect stronger negative responses for forest ground herbs than for ferns. Alternatively, lack of support for this proposition could indicate that, besides dispersal, other species’ characteristics are determining species’ responses to fragmentation [12]. For instance, in the case of forest herbs, it can be speculated that capabilities such as the ability to form persistent populations through extended and prolonged clonal growth or by producing long term persistent seeds [20] may facilitate the permanence of herb species in forest fragments and thus offset potential negative effects of fragmentation. Similarly, variations in shade tolerance across forest herb and fern species, and hence, in their respective possibilities to survive in more open habitats (e.g. forest edges) may result in a range of responses to fragmentation, thus impeding our ability to detect differences between them associated with their dispersal capabilities. However, information about species’ shade tolerance and other potentially relevant traits is rare for forest ground species and this prevents us from formulating straightforward predictions in those cases. Thus, in this study we first test the reaction of each species to fragmentation and then propose potential explanations based on what is known about the studied species.

Here we present a broad-scale, grid-based analysis (cell size = 10 x 10 km) of the relationships between occurrences of 16 forest understory plant species and forest amount and fragmentation. For each unit of analysis we quantify both its percentage of forest cover (our habitat amount variable), and the proportion of this percentage occurring in the largest forest patch (our forest fragmentation variable). This landscape approach has been previously used in theoretical [7] and empirical studies [12, 23] and allows estimating species’ occurrence probabilities along the forest cover gradient for different levels of fragmentation, and to test directly the fragmentation threshold hypothesis. We specifically evaluate: a) the degree of sensitivity of each species to forest loss and fragmentation, and b) if fragmentation is related to extinction thresholds. We discuss our results in a species-specific trait context.

## Methods

### Plant data

Data on the distributions of herb and fern species were extracted from various published sources [24–26], and two web resources, the Anthos project (www.anthos.es; accessed January, 2013) and the SIVIM project (www.sivim.info; accessed January, 2013), both describing the presences and absences of Iberian vascular plants in the 10 x 10 km Universal Transverse Mercator (UTM) grid system. Our analysis units were the cells of the grid covering Peninsular Spain, although we excluded all coastal cells covering < 50% of the land mass of inland cells, which rendered a total of 5,279 cells for the study. Information included in Anthos and SIVIM databases has been collected from floristic collections, inventories, atlases, doctoral theses and research articles. At the dates we accessed them, Anthos included over 15,000 references whereas SIVIM had compiled over 130,000 vegetation inventories (i.e., around 2,100,000 floristic records). Accordingly, we accept that vascular plants of the Iberian Peninsula are rather well sampled and, hence, we assume that our presence/absence database is quite accurate. On the other hand, it should be noted that most of the information included in Anthos and SIVIM was gathered during the decades of the 80s and 90s of the 20th century; that is, during a time period just preceding the moment in which the source of our forest habitat data, the CORINE Land Cover 2000 database (described in next section), was generated. Thus, although the temporal matching between our species occurrence and forest habitat data is not perfect, we are confident that it is enough to provide robust results on the relationships between both types of variables, particularly bearing in mind the relatively slow dynamics of forest perennials and their forest habitats, and both the broad geographical extent of our study and the coarse resolution of the analysis units we used.

We studied forest specialist herbs and ferns defined in the literature as “shade-tolerant” that primarily occur in the understory of core forest habitat conditions or ancient forests [27, 28]. We investigated the impacts of forest loss and fragmentation on each species focusing on its native range in the study area (i.e. the presences/absences of each species were analyzed using the set of cells that overlapped its native range in Peninsular Spain). We identified the cells corresponding to each native range by digitizing species’ range maps included in Bolòs and Vigo [29] and Salvo [30] and overlaying them on our grid. Of the forest-specialist species included in the data set, we selected 10 herbs and six ferns that were not under or over-represented within their ranges (i.e. present in >15% and <70% of their cells, respectively) (Table 1). Species not meeting this criterion were excluded as both statistical models and their evaluation tests may not be reliable (see [31] and references therein).

**Table 1:** List of the 16 forest understory herbs and ferns included in the study.

Plant group/ family	Scientific name	Common name	Percentage of presences within native range	Native range size (number of cells)
Herbs				
Ranunculaceae	*Helleborus viridis* L.	Green hellebore	53.4	380
Poaceae	*Poa nemoralis* L.	Wood meadow-grass	38.5	1571
Lamiaceae	*Teucrium scorodonia* L.	Wood sage	33.8	1452
Saxifragaceae	*Saxifraga hirsuta* L.	Kidney saxifrage	31.4	516
Ranunculaceae	*Hepatica nobilis* Schreb.	Common hepatica	29.4	1174
Poaceae	*Deschampsia flexuosa* L.	Wavy hair-grass	25.1	1382
Ericaceae	*Monotropa hypopitys* L.	Yellow bird's-nest	20.4	709
Melanthiaceae	*Paris quadrifolia* L.	Herb-paris	19.4	603
Papaveraceae	*Meconopsis cambrica* L.	Welsh poppy	19.4	329
Paeoniaceae	*Paeonia broteri* Boiss. & Reut.	Broteroi peony	16.5	1809
Ferns				
Blechnaceae	*Blechnum spicant* (L.) Roth	Hard fern	39.2	1131
Dryopteridaceae	*Polystichum aculeatum* (L.) Roth	Hard Shield-fern	34.7	869
Dryopteridaceae	*Dryopteris filix-mas* (L.) Schott	Common Male fern	33.5	1477
Woodsiaceae	*Athyrium filix-femina* (L.) Roth	Lady fern	31.8	1569
Aspleniaceae	*Asplenium onopteris* L.	Irish spleenwort	28.8	2432
Dryopteridaceae	*Dryopteris dilatata* (Hoffm.) A. Gray	Broad Buckler-fern	18.3	610

For each plant group, the species have been ranked according to decreasing occurrence frequencies within their native ranges in Peninsular Spain (expressed as percentages of occurrences in UTM cells of 10x10 km each covering the species’ native distributions in the study area). Native range sizes are also provided. Species nomenclature follows Tutin et al. [58].

### Forest variables

A previous analysis found that the proportion of forest amount varies independently of climate, topography, soil and major perturbation events in Spain [32], and thus we only included forest variables in this study. Specifically, we used the CORINE Land Cover database 2000 (CLC2000) to quantify forest amount and fragmentation. CORINE Land Cover is a satellite imagery-based land cover classification that provides consistent information on land cover across Europe, of which we utilized the 100-m pixel resolution (all data available at: http://www.eea.europa.eu/data-and-maps/data/corine-land-cover-2000-clc2000-seamless-vector-database-3; accessed January, 2013). Of the 44 land-cover classes recognized by this database, we focused on the three reflecting forest cover (broadleaved, coniferous and mixed forests) and classified all pixels belonging to them as forest habitat. Next, we superimposed the 10 x 10 km grid and computed forest amount and fragmentation for each cell; namely, the percentage of each cell’s area covered by forest [percentage forest cover (*PFC*)], and the percentage of this forest cover that corresponds to the largest forest patch [relative largest patch size (*rLPS*)]. The latter can be used to distinguish between scenarios reflecting low and high fragmentation. Briefly, for any given value of *PFC*, weakly fragmented cells would be those with most forest clustered into a single patch; that is, with high *rLPS*, as the remaining forest will be too limited to constitute many additional patches. In contrast, for the same value of *PFC*, strongly fragmented cells would be those with very small *rLPS*, as this implies that the other patches would be even smaller and in general more numerous than in the former case [23].

### Numerical methods

#### Species distribution modeling

We used logistic regression for modeling the relationship between the probability of occurrence (*p*) of each species and forest cover (*PFC*) and fragmentation (*rLPS*) as:

$$p\;\text{=}\;\exp(\beta_{\text{0}}\text{+}\beta_{\text{1}}PFC\text{+}\beta_{\text{2}}rLPS)/1+\exp(\beta_{\text{0}}\text{+}\beta_{\text{1}}PFC\text{+}\beta_{\text{2}}rLPS)$$ (1)

where *β*_0_ is the model intercept and *β*_1_ and *β*_2_ are the slopes of *PFC* and *rLPS*, respectively. The collinearity of the independent variables was low for all models with VIF (Variance Inflation Factor) values lower than 1.34. We evaluated the performance of each model according to its Chi-square’s *p*-value and two additional goodness of fit measures, the Area Under the Receiver Operating Characteristic Curve (AUC) and the McFadden’s rho squared (*ρ* ^2^). We wanted to test the fragmentation threshold hypothesis (see below) using robust models, for which we excluded all species whose models did not have a statistically significant Chi-square (p<0.05), and AUC and *ρ* ^2^ values greater than 0.7 and 0.2, respectively (see [12] for more details on these threshold levels). Logistic regression modeling was done in Matlab (MathWorks Inc., version 7.0), *ρ* ^2^ values were computed following McFadden [33], and AUC values using the 'PresenceAbsence' package [34] implemented in R (R Development Core Team 2008).

#### Sensitivity analysis to forest amount and fragmentation

Following Rueda et al. [12], we used parameterized logistic models to generate scalar metrics of species' sensitivities to changes in both forest cover in absence of fragmentation (Ω_*j*,*cover*_), and fragmentation at a constant forest cover (Ω_*j*,*fragm*_). For the zero fragmentation scenario (i.e. with *rLPS* = 100%) we computed the proportional reduction in occurrence due to a reduction in forest cover from a high (*PFC =* 75%) to a low value (5%):

$$\Omega_{j,cover}=ln[p(j,PFC=5,rLPS=100)/p(j,PFC=75,rLPS=100)]$$ (2)

where a positive or negative Ω_*j*,*cover*_ implies that the species *j* responds positively or negatively to forest contraction.

**Fig 1:**
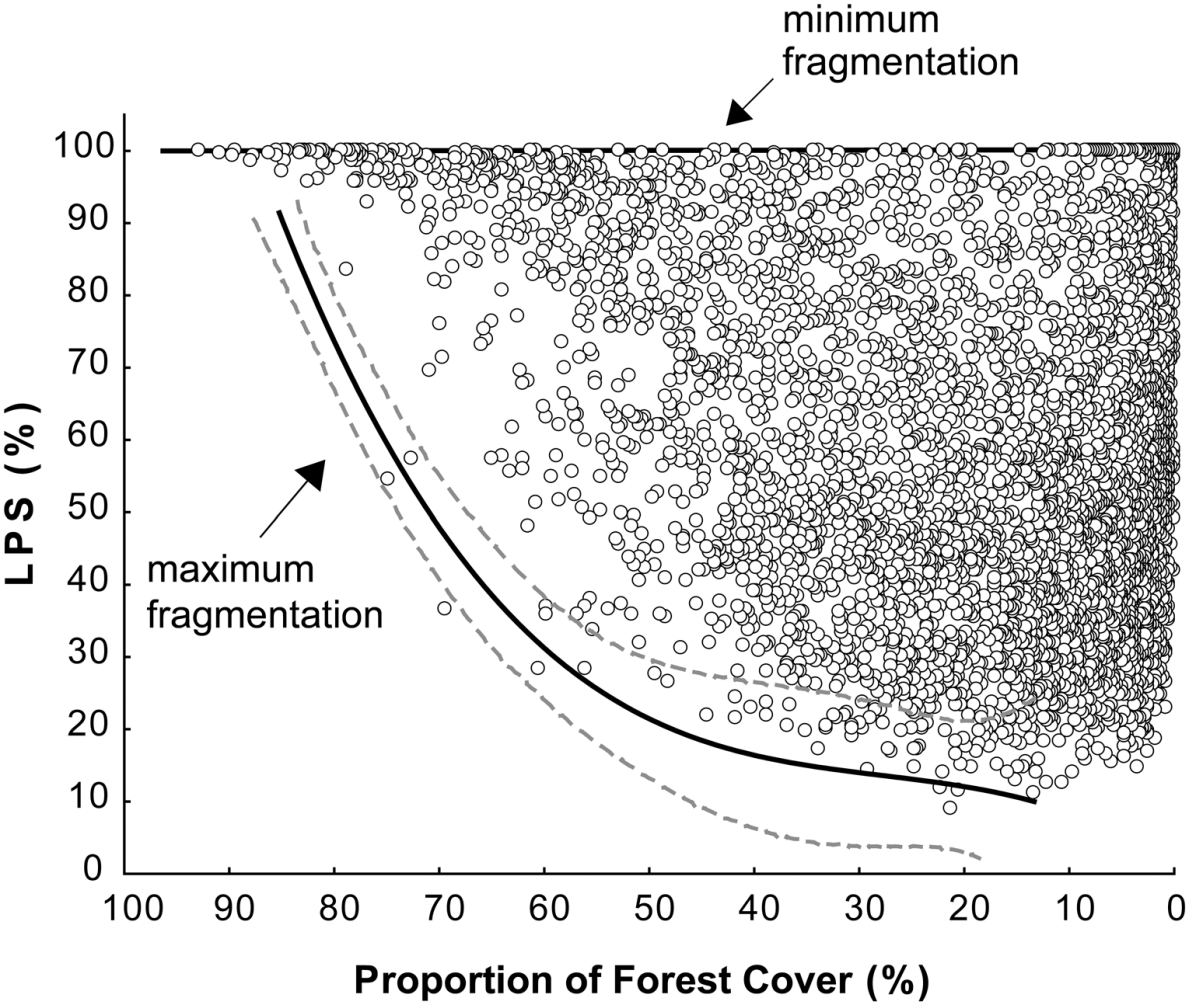
Forest fragmentation as a function of the proportion of forest cover *PFC* in 10 x 10 km cells in Peninsular Spain. Forest fragmentation is quantified as the percentage of forest occurring in the largest patch, *rLPS*. The upper solid line delimitates cells with near zero fragmentation (*rLPS ≈*100%). The lower solid and discontinuous lines (95% CIs) correspond to a polynomial regression on 100 cells located at the lower edge of the cloud of points. We used this line to quantify maximum fragmentation in terms of *rLPS* at varying levels of *PFC* (see text). Note that *PFC* decreases to the right.

For the scenario of constant forest cover, we first examined the relationship between *rLPS* and the proportion of forest cover (Fig 1) and observed that cells with near zero fragmentation (*rLPS ≈*100%) occur throughout the forest cover gradient, but differences between minimum and maximum fragmentation (indicated by higher and lower *rLPS* values, respectively, Fig 1) increase as forest cover contracts. Based on these empirical relationships, we focused on a moderately low forest amount (*PFC* = 20%) and computed the proportional reduction in occurrence due to an increase from zero to maximum fragmentation at this forest cover (i.e. for the *rLPS* values of 100 and 10, respectively):

$$\Omega_{j,fragm}=ln[p(j,PFC=20,rLPS=10)/p(j,PFC=20,rLPS=100)]$$ (3)

where a negative Ω_*j*,*fragm*_ indicates a negative response of species *j* to increased fragmentation, whereas a positive Ω_*j*,*fragm*_ indicates that fragmentation favors the occurrence of species.

We generated 95% Confidence Intervals (CIs) for Ω_*j*,*cover*_ and Ω_*j*,*fragm*_ using a bootstrap procedure implemented in Matlab (MathWorks Inc., version 7.0). We produced 1,000 bootstrap samples for each species by resampling with replacement triads of *PFC*, *rLPS* and species occurrence (1 or 0) values from each original sample. Each bootstrap sample comprised the same number of cells as the original sample and was used to parameterize the logistic model (Eq 1) and to compute Ω_*j*,*cover*_ and Ω_*j*,*fragm*_ as in Eqs 2 and 3, respectively. With this procedure we generated 1,000 ranked values per scalar metric, of which the values at the 25th and 976th positions corresponded to the lower and upper limits of the metric's 95% CI. Finally, we calculated the mean for Ω_*j*,*cover*_ and Ω_*j*,*fragm*_ for herbs and ferns to assess differences in sensitivity to forest loss (zero fragmentation scenario) and fragmentation (constant forest amount scenario). To obtain the 95% CIs for the means, we calculated 1,000 bootstrap mean Ω_*j*,*cover*_ and Ω_*j*,*fragm*_ for the species of each group as explained above.

#### Testing the extinction threshold hypothesis

Ideally, the extinction threshold of a particular species would be reached when its occurrence probability equals zero [1]. However, this is of no use when using logistic regression models, as a probability of 0 (and 1) is approached asymptotically but never attained. As an alternative, Woodroffe and Ginsberg [35] focused on a logistic probability of 0.5 (i.e. at which the species presence or absence become equally likely) in their investigation of carnivore extinction and reserve size, and Rueda et al. [12] did the same to investigate fragmentation thresholds in European forest birds. We adopted the same criterion, and thus tested for the possibility that the forest cover level at which a species reaches an occurrence probability of 0.5 was higher under maximum forest fragmentation.

We used the model parameterized for each species to compute its occurrence probability in two extreme fragmentation scenarios, maximum and zero at nine levels of forest cover (*PFC* values of 85, 75, 65, 55, 45, 35, 25, 15 and 5%), all of them occurring within the range of original *PFC* values observed for each species. We defined maximum fragmentation levels based on the existing empirical relationship between *PFC* and *rLPS* (see Fig 1). First, we selected 100 cells that delimited the cloud of points in Fig 1 and that represent higher fragmentation at different forest covers. Next, we computed a fourth order polynomial regression of *rLPS* on *PFC* with these cells and used this model to obtain the *rLPS* that approximates the maximum empirical fragmentation for each of the nine forest covers. This gave *rLPS* values of 90, 60, 40, 26, 20, 16, 14, 10 and 5%. Again, these *rLPS* values occur within the range of original *rLPS* values observed for each species. Then, we calculated the probability of occurrence of each species using the nine levels of *PFC* and the *rLPS* values; namely (85,90), (75,60), (65,40). (5,5). For the zero fragmentation scenario, we assumed that *rLPS* = 100% (i.e. that all forest is clustered in one patch) and computed the occurrence probabilities for the corresponding *PFC* and *rLPS* pairs; namely (85,100), (75,100), (65,100). (5,100). Statistical differences between the extinction thresholds found for each fragmentation scenario were again established based on bootstrapped 95% CIs.

## Results

Of the 16 forest understory species modeled, robust models meeting all three goodness of fit criteria were obtained for nine, comprising five herbs (*Monotropa hypopitys*, *Hepatica nobilis*, *Teucrium scorodonia*, *Poa nemoralis* and *Deschampsia flexuosa*), and four ferns (*Polystichum aculeatum*, *Dryopteris filix-mas*, *Athyrium filix-femina* and *Blechnum spicant*) (Table 2).

**Table 2:** Goodness-of-fit statistics for the forest cover and fragmentation logistic models generated for ten herbs and six ferns inhabiting forest understories in Peninsular Spain.

Plant group/	Goodness-of-fit of logistic regression model		
Species	Chi-squared *p*	*ρ* ^*2*^	*AUC*
Herbs			
* * ***Poa nemoralis***	<0.0001	0.22	0.81
* * ***Monotropa hypopitys***	<0.0001	0.21	0.81
* * ***Hepatica nobilis***	<0.0001	0.21	0.78
* * ***Deschampsia flexuosa***	<0.0001	0.21	0.76
* * ***Teucrium scorodonia***	<0.0001	0.20	0.75
* Helleborus viridis*	<0.0001	0.16	0.73
* Paeonia broteri*	<0.0001	0.05	0.70
* Saxifraga hirsuta*	<0.0001	0.07	0.68
* Paris quadrifolia*	<0.0001	0.04	0.64
* Meconopsis cambrica*	0.004	0.03	0.63
Ferns			
* * ***Dryopteris filix-mas***	<0.0001	0.22	0.79
* * ***Blechnum spicant***	<0.0001	0.21	0.77
* * ***Athyrium filix-femina***	<0.0001	0.20	0.77
* * ***Polystichum aculeatum***	<0.0001	0.20	0.76
* Asplenium onopteris*	<0.0001	0.13	0.73
* Dryopteris dilatata*	<0.0001	0.11	0.72

For each plant group, the species have been ranked according to decreasing AUC* values. The logistic models of the first five seed plants and four ferns (in bold) met all three goodness-of-fit criteria described in the Methods (i.e. a Chi-squared’s *p*-value <0.05, AUC ≥ 0.7 and McFadden’s *ρ* ^*2*^ ≥ 0.2) and were selected for testing the fragmentation threshold hypothesis.

*AUC = Area Under the Receiver Operating Characteristic Curve

### Sensitivity to forest amount *per se* and fragmentation *per se*

**Fig 2:**
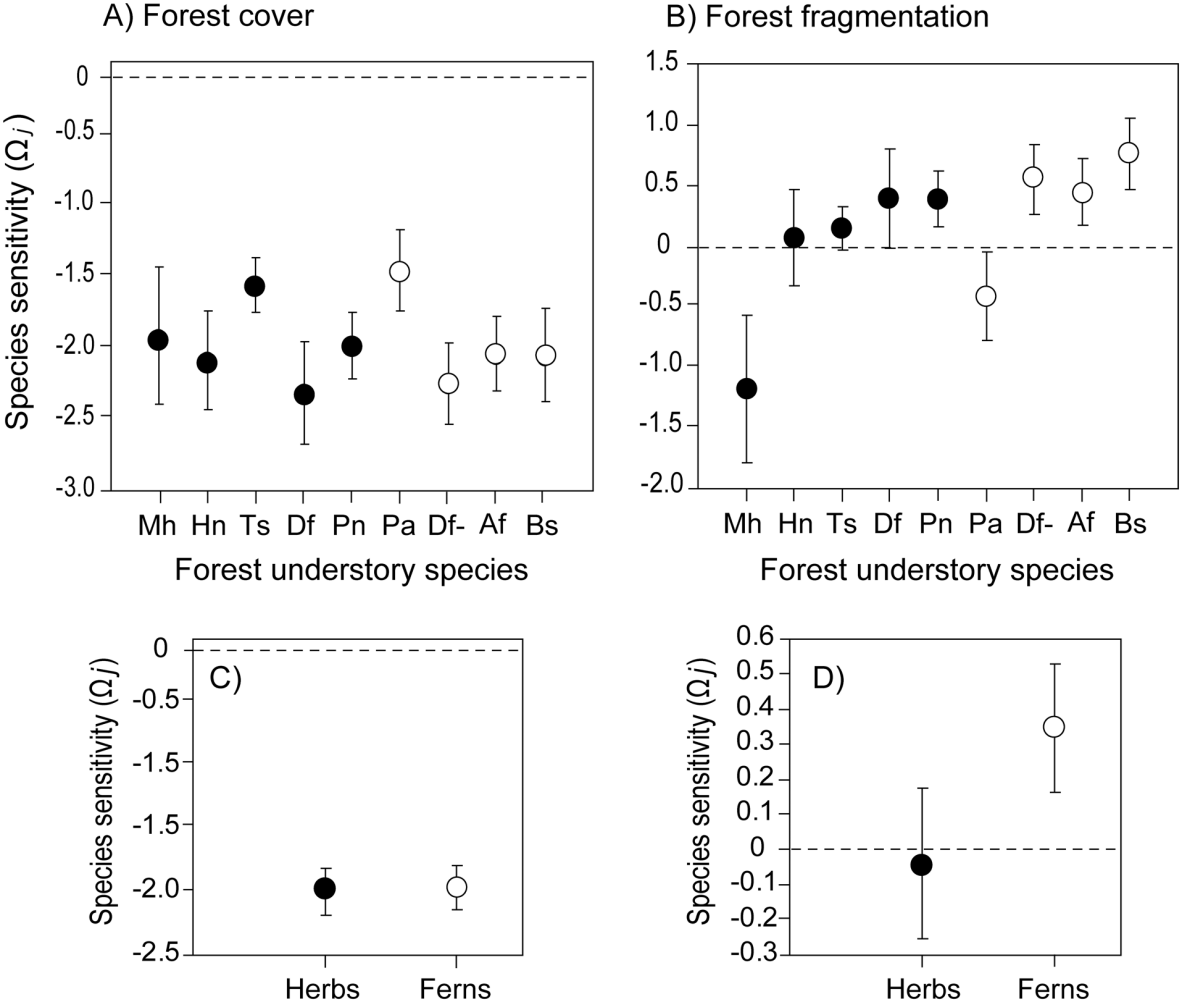
Sensitivity Ω_*j*_ (± 95%) of five herbs (black circles) and four ferns (white circles) to forest cover contraction in absence of fragmentation (A) and forest fragmentation increases at constant forest cover (B). Species are considered significantly sensitive to forest cover contraction or forest fragmentation when 95% CIs do not overlap with zero. Mh, *Monotropa hypopitys*; Hn, *Hepatica nobilis*; Ts, *Teucrium scorodonia*; Df, *Deschampsia flexuosa*; Pn, *Poa nemoralis*; Pa, *Polystichum aculeatum*; Df-, *Dryopteris filix-mas*; Af, *Athyrium filix-femina*; Bs, *Blechnum spicant*. C) Average Ω_*j*_ (± 95%) response of herbs (black circle) and ferns (white circle) to forest reduction and D) to fragmentation. Note that the logarithm scale generates a non-linear relationship between Ω_*j*_ with the proportional reduction in occurrence. For example, Ω_*j*,_ = -3 would imply a 95% reduction in occurrence; Ω_*j*,_ = -1 a 63% reduction; Ω_*j*,_ = -0.5, a 39% reduction, and Ω_*j*,_ = +1 would imply a 170% increase in occurrence.

As expected for forest understory plants, all species responded negatively to forest cover contraction in absence of fragmentation (a negative Ω_*j*,*cover*_ with 95% CIs not including zero) (Fig 2A). In contrast, for the scenario of increased fragmentation, only two species showed negative Ω_*j*,*fragm*_ values with 95% CIs not overlapping zero (Fig 2B). These species were thus significantly negatively affected by fragmentation and comprised one herb (*M*. *hypopitys*) and one fern (*P*. *aculeatum*), although the latter had a weaker response as indicated by the close proximity of its upper 95% CI to zero. Additionally, five species, the herbs *P*. *nemoralis* and *D*. *flexuosa*, and three ferns (*D*. *filix-mas*, *A*. *filix-femina*, and *B*. *spicant*) responded positively and, hence, are more likely to be found in more fragmented cells. Again, *D*. *flexuosa* had a weaker response as indicated by the close proximity of its lower 95% CI to zero. The occurrence of two herbs was not significantly affected by fragmentation (*H*. *nobilis*, and *T*. *scorodonia*). As groups, herbs and ferns were equally affected by forest contraction (Fig 2C). Ferns were, on average, favored by fragmentation, and herbs did not show sensitivity to fragmentation different from zero (Fig 2D).

### Fragmentation and extinction thresholds

**Fig 3:**
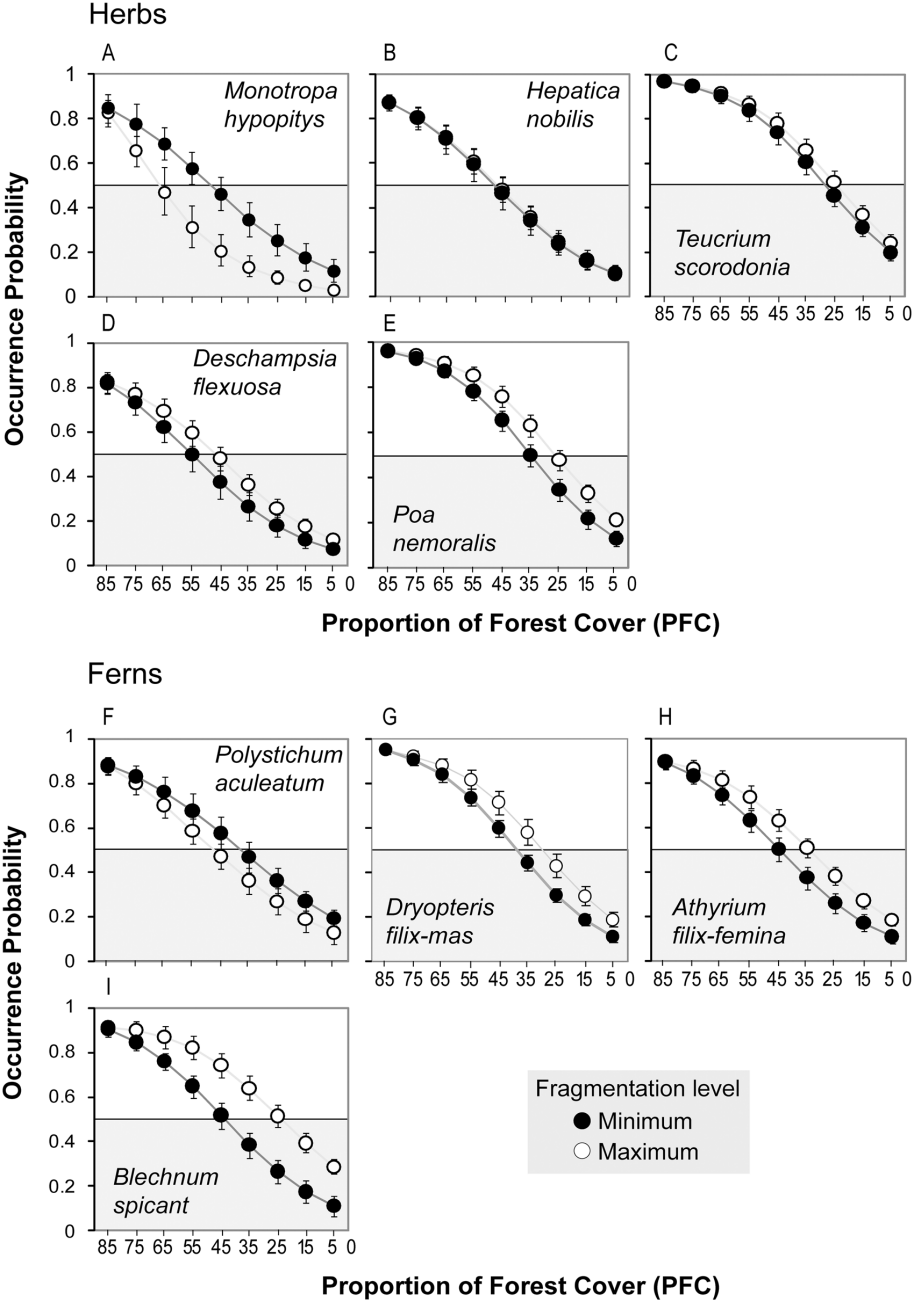
Occurrence probability of the five herbs and four ferns under the two scenarios of forest fragmentation. The nine predictive values of forest cover (*PFC*) range from 5% to 85%. White circles represent occurrence probabilities in the maximum fragmentation scenario, while black circles represent occurrence probabilities in the minimum fragmentation scenario. Confidence intervals at 95% are given. Note that *PFC* decreases to the right.

Consistent with the sensitivity analysis, the occurrence probabilities of all nine species decreased with forest cover reduction in both scenarios, indicating that all were negatively affected by forest cover loss (Fig 3). To test the fragmentation threshold hypothesis, we focused on where, in the gradient of forest cover reduction, the first overlap between the 95% CI of an occurrence probability and the critical probability of 0.5 occurred, which, according to the fragmentation hypothesis, should occur at a higher forest cover under maximum fragmentation. This was the case for the non-photosynthetic, mycorrhizal herb *M*. *hypopitys* and the fern *P*. *aculeatum*, whose 95% CIs first overlapped the critical probability of 0.5 at forest covers of 65% and 45% under maximum fragmentation, and at 55% and 35% under no fragmentation, respectively (see Fig 3A and 3F). Apart from supporting the fragmentation threshold hypothesis, these results again suggest a stronger sensitivity to increased forest loss and fragmentation for *M*. *hypopitys*, as this species first reached the 0.5 probability at a higher forest cover in each fragmentation scenario and also showed significantly different occurrence probabilities between both scenarios at all forest covers except for the highest (*PFC* = 85%, see Fig 3A). In contrast, for *P*. *aculeatum* these significant differences only occurred at intermediate *PFC* values (see Fig 3F). On the other hand and consistent with the sensitivity analysis, there were two herbs that showed no significantly different extinction thresholds between both fragmentation scenarios, *H*. *nobilis* and *T*. *scorodonia* (see Fig 3B and 3C), and five species that exhibited the opposite response to that predicted by the fragmentation hypothesis (i.e. a significantly higher extinction threshold under no fragmentation): the herbs *D*. *flexuosa* and *P*. *nemoralis*, and the ferns *A*. *filix-femina*, *D*. *filix-mas* and *B*. *spicant* (Fig 3D,3E and 3G–3I).

Taken together, these results suggest that support for the fragmentation threshold hypothesis is rare among forest understory plant species, and also that some of these species may even react positively to increased fragmentation. Moreover, herbs do not show negative responses to fragmentation, i.e., our expectation based on lower dispersal abilities of forest herbs was not supported.

## Discussion

The array of responses to fragmentation showed by the modeled species underscore the complexity that interacting ecological processes associated with fragmentation may have on specific species [13]. Lack of support for our expectation based on the low dispersal capability of forest herbs could indicate that other factors contribute to species’ responses to fragmentation [28]. We suggest that variation in shade tolerance across species, and hence, in their respective possibilities to survive in more open, fragmented habitats (e.g. in forest edges) may likely result in a range of responses to fragmentation that preclude finding clearer patterns.

Two species, one herb (*Monotropa hypopitys*) and one fern (*Polystichum aculeatum*) supported the predictions of the fragmentation threshold hypothesis. *M*. *hypopitys*, unlike most plants, does not contain chlorophyll, and acquires carbohydrates from trees through a parasitic symbiosis with *Trichola* fungi species [36, 37]. Its lack of dependence on light makes *M*. *hypopitys* clearly specialized to live in dark areas such as dense forest floors and may, in part, explain why this species was highly sensitive to forest fragmentation (Figs 2B and 3A). This could help explain the marked decline in the abundance of *M*. *hypopitys* in many parts of Europe [38]. *Polystichum aculeatum* has, as do the majority of ferns, the ability to quickly colonize new sites and to spread rapidly over great distances [39]. However, Carranza et al. [40] found that, contrary to other ferns (e.g., *Dryopteris filix-mas*) whose frequency is higher in fragmented forests, *P*. *aculeatum* is present exclusively in medium and non-fragmented forests, and they attributed this to the low capacity of this fern to tolerate high levels of light. The other three fern species (*Dryopteris filix-mas*, *Athyrium filix-femina*, *Blechnum spicant*) are well known for their great ecological amplitude and colonization capability [41–43], life history traits that can be expected to allow them to escape from the negative processes associated with fragmentation (Fig 2B, 2G and Fig 3G–3I). It is likely that these three fern species are also more generalist in their niche requirements, thus they may be able to better cope with changing landscape structures [44]. However, the fact that ferns are much less well studied than angiosperms, and much of our knowledge on them comes from rather general ecological studies, makes drawing conclusions about fern responses to fragmentation a challenge.

Several lines of evidence suggest that the herbs studied here are not as shade-tolerant as *M*. *hypopitys*. Thus, for example, although several traits that characterize *Hepatica nobilis* (e.g. seeds dispersed by ants [28], and absence of clonal growth or seed bank [45]) make this species a candidate for negative impacts of fragmentation, it has been observed that increased shade from trees may impair its flowering and cause considerable declines in its populations, and also that the species is able to survive in open wooded meadows [46]. This may explain why this species shows null responses to fragmentation (Fig 3B). *Teucrium scorodonia* and *Deschampsia flexuosa* have also been described in hedges, heaths and grasslands—that probably owe their origin to the clearance of original woodland—in Britain [47, 48] and the Iberian Peninsula [49, 50]. Also, germination of both species is indeed less successful in the shade/closed canopy compared with sunny conditions [48, 51]. Further, *Poa nemoralis*, one of the species clearly favored by forest fragmentation (Fig 3E), has a germination rate of up to 60% in the sun in contrast to 7% in the shade [52], and when this species occurs in the densest canopy forest cover is in the form of scattered and sterile individuals [51]. Also, this species is effectively dispersed in animal fur or in deer dung [53, 54], which would facilitate the colonization of forest fragments [55]. All of these characteristics suggest that, although able to survive in dense canopy forests, these herbs grow and reproduce better in forests with sparse canopy, forest gaps or even forest edges.

Together with the typical problems of empirical studies, e.g. to use a general definition of habitat for all species (forest), our analysis entails several limitations that might make unclear the response of the species in some cases. Our database comprises species presence/absence in 10 km x 10 km grid cells, which can be too coarse a resolution to detect some fragmentation effects. Thus, we lack information about the processes acting at the landscape level, namely demographic processes such as source-sink dynamics that are not captured by presence/absence data. However, using species densities, Montoya et al. [23] only confirmed the extinction threshold hypothesis for one out of eight tree species, which may suggest that, in general, forest species may show a weak response to fragmentation due to their own idiosyncrasy (e.g. long-lived perennials). Additionally, from the wealth of existing metrics quantifying habitat configuration and landscape matrix characteristics (e.g. measuring patch densities and sizes, habitat connectivity and contrast levels, and distances between patches), we chose to measure fragmentation with a single, easily interpretable index (*rLPS*), and therefore we could be missing information about species’ responses to changing landscape configuration (e.g. [56]; but see [57]). Further analyses might consider these issues by including higher resolution scales, additional fragmentation metrics and data on landscape matrix characteristics. Finally, another potential concern is related to the reliability of our data, particularly to that of species absences. As it is well known, species absences are typically more difficult to quantify accurately, which could in turn have affected the consistency of our logistic models and our conclusions. We, however, are very confident that our data are extremely robust in this regard for two main reasons. Firstly because the Iberian Peninsula (as it is common in other western European regions such as France) has a long tradition of vegetation studies and has been particularly well surveyed floristically, as reflected by e.g. the SIVIM database (one of the two main data sources we used, see Methods), which includes over 130,000 plant community inventories distributed across this region. And secondly because any potential impact of varying species detectability or sampling intensities that could exist in the databases we processed are likely to have been minimized by the relatively coarse resolution of our analysis units (i.e., 10-km cells).

In sum, we have empirically identified fragmentation extinction thresholds for two out of nine forest understory species, suggesting that fragmentation extinction thresholds are uncommon among forest understory plant species. Yet, these results, together with those obtained by Rueda et al. [12]—who following the same methodology found fragmentation extinction thresholds among forest birds—reinforce the existence of fragmentation-driven thresholds for certain forest specialist species. We highlight the importance of focusing on the type of species that are more prone to extinction in fragmented landscapes since neglecting adverse fragmentation effects for them can lead these species to local extinction. A more complete knowledge about forest species traits can help in this task.

## References

[1] Fahrig L. Effect of habitat fragmentation on the extinction threshold: a synthesis. Ecol Appl. 2002; 12: 346–353.

[2] Haila Y. A conceptual genealogy of fragmentation research: from island biogeography to landscape ecology. Ecol Appl. 2002; 12: 321–334.

[3] Fahrig L. Effects of habitat fragmentation on biodiversity. Annu Rev Ecol Syst. 2003; 34: 487–515.

[4] Fischer J, Lindenmayer DB. Landscape modification and habitat fragmentation: a synthesis. Global Ecol Biogeogr. 2007; 16: 265–280.

[5] Secretariat of the Convention on Biological Diversity. Global Biodiversity Outlook 3; 2010. Available: http://www.cbd.int/doc/publications/gbo/gbo3-final-en.pdf.

[6] Didham RK, Kapos V, Ewers RM. Rethinking the conceptual foundations of habitat fragmentation research. Oikos 2012; 121: 161–170.

[7] Bascompte J, Solé RV. Habitat fragmentation and extinction thresholds in spatially explicit models. J Anim Ecol. 1996; 65: 465–473.

[8] Hanski I, Moilanen A, Gyllenberg M. Minimum viable metapopulation size. Am Nat. 1996; 147: 527–541.

[9] Lande R. Extinction thresholds in demographic models of territorial populations. Am Nat. 1987; 130: 624–635.

[10] Betts MG, Graham JF, Diamond AW. Thresholds in Songbird Occurrence in Relation to Landscape Structure. Conserv Biol. 2007; 21: 1046–1058. 17650254 10.1111/j.1523-1739.2007.00723.x

[11] Zuckerberg B, Porter WF. Biological thresholds in the long-term responses of breeding birds to forest cover and fragmentation. Biol Conserv. 2010; 143: 952–962.

[12] Rueda M, Hawkins BA, Morales-Castilla I, Vidanes R, Ferrero M, Rodríguez MA. Does fragmentation increase extinction thresholds? A European-wide test with seven forest birds. Global Ecol Biogeogr. 2013; 22: 1282–1292.

[13] Betts MG, Fahrig L, Hadley AS, Halstead JB, Robinson WD, Wiens JA, et al A species-centered approach for uncovering generalities in organism responses to habitat loss and fragmentation. Ecography 2014; 37: 517–527.

[14] Lindenmayer DB, Fischer J. Tackling the habitat fragmentation panchreston. Trends Ecol Evol. 2006; 22: 127–132. 17145095 10.1016/j.tree.2006.11.006

[15] Angelstam P. Conservation of communities—the importance of edges, surroundings and landscape mosaic structure In: Hansson L editor. Ecological principles of nature conservation. Springer US; 1992 pp. 9–70.

[16] Andrén H. Effects of landscape composition on predation rates at habitat edges In: Hansson L, Fahrig L, Merriam G, editors. Mosaic landscapes and ecological processes. Springer Netherlands; 1995 pp. 225–255.

[17] Hanski I. Effects of landscape pattern on competitive interactions In: Hansson L, Fahrig L, Merriam G, editors. Mosaic landscapes and ecological processes. Springer Netherlands; 1995 pp. 204–224.

[18] Honnay O, Jacquemyn H, Bossuyt B, Hermy M. Forest fragmentation effects on patch occupancy and population viability of herbaceous plant species. New Phytol. 2005; 166: 723–736. 15869637 10.1111/j.1469-8137.2005.01352.x

[19] Atlegrim O, Sjöberg K. Response of bilberry (*Vaccinium myrtillus*) to clearcutting and single-tree selection harvests in uneven-aged boreal *Picea abies* forests. Forest Ecol Manag. 1996; 86: 39–50.

[20] Godefroid S, Rucquoij S, Kpedam N. To what extent do forest herbs recover after clearcutting in beech forest? Forest Ecol Manag. 2005; 210: 39–53.

[21] Kessler M. Biogeography of ferns In: Mehltreter K, editor. Fern Ecology. Cambridge University Press; 2010 pp. 22–60.

[22] Aranda SC, Gabriel R, Borges PA, Santos A, Hortal J, Baselga A, et al How do different dispersal modes shape the species-area relationship? Evidence for between-group coherence in the Macaronesian flora. Global Ecol Biogeogr. 2013; 22: 483–493.

[23] Montoya D, Albuquerque FS, Rueda M, Rodríguez MA. Species’ response patterns to habitat fragmentation: do trees support the extinction threshold hypothesis? Oikos 2010; 119: 1335–1343.

[24] Izuzquiza A. Mapa 58 *Monotropa hypopitys* L. Asientos para un Atlas Corológico de la Flora Occidental 8. Fontqueria 1988; 17: 1–36.

[25] Villar L, Sesé JA, Ferrández JV, Saule M. Atlas de la flora del Pirineo Aragonés. 2 Vols Huesca: Consejo de Protección de la Naturaleza de Aragón e Instituto de Estudios Altoaragoneses; 1997–2001.

[26] Bolòs O. Atlas corològic de la flora vascular dels Països Catalans Primera compilació general. 2 Vols Barcelona: Institut d’Estudis Catalans, Barcelona; 1998.

[27] Honnay O, Degroote B, Hermy M. Ancient-forest plant species in western Belgium: a species list and possible ecological mechanism. Belg J Bot. 1998; 130: 139–154.

[28] Hermy M, Honnay O, Firbank L, Grashof-Bokdam C, Lawesson JE. An ecological comparison between ancient and other forest plant species of Europe, and the implications for forest conservation. Biol Conserv. 1999; 91: 9–22.

[29] Bolòs O, Vigo J. Flora dels Països Catalans. Vol. I-IV Barcelona: Barcino; 1984–2001.

[30] Salvo E. Guía de helechos de la Península Ibérica y Baleares. Pirámide; 1990.

[31] Jiménez-Valverde A, Lobo JM, Hortal J. Not as good as they seem: the importance of concepts in species distribution modelling. Divers Distrib. 2008; 14: 885–90.

[32] Montoya D, Zavala MA, Rodríguez MA, Purves DW. Animal *versus* wind dispersal and the robustness of tree species to deforestation. Science 2008; 320: 1501–1504. 10.1126/science.1158404 18535208

[33] McFadden D. Quantitative methods for analyzing travel behaviour of individuals: some recent developments In: Hensher DA, Stopher PR, editors. Behavioural Travel Modelling. Croom Helm; 1979 pp 279–318.

[34] Freeman EA, Moisen G. PresenceAbsence: an R package for presence absence analysis. J Stat Software 2008; 23: 1–31.

[35] Woodroffe R, Ginsberg JR. Edge effects and the extinction of populations inside protected areas. Science 1998; 280: 2126–2128. 9641920 10.1126/science.280.5372.2126

[36] Björkman E. *Monotropa hypopitys* L. and epiparasite on tree roots. Physiol Plantarum 1960; 13: 308–327.

[37] Leake JR, McKendrick S, Bidartondo M, Read DJ. Symbiotic germination and development of the myco-heterotroph *Monotropa hypopitys* in nature and its requirement for locally distributed Tricholam spp. New Phytol. 2004; 163: 405–423. 10.1111/j.1469-8137.2004.01115.x 33873615

[38] Beatty G, Provan J. High clonal diversity in threatened peripheral populations of the yellow bird's nest (*Hypopitys monotropa*; syn. *Monotropa hypopitys*). Ann Bot 2011; 107: 663–670. doi: 10.1093/aob/mcr003 21257715 10.1093/aob/mcr003 PMC3064538

[39] Mütter H, Birksand HJB, Odland A. The comparative ecology of *Polystichum aculeatum*, *P*. *braunii*, and *P*. *lonchitis* in Hordaland, western Norway. Nord J Bot. 1998; 18: 267–288.

[40] Carranza ML, Frate L, Paura B. Structure, ecology and plant richness in fragmented beech forests. Plant Ecol Diver. 2012; 5: 541–551.

[41] Corley HV. Dryopteris filix-mas agg. Britain. Proc Bot Soc Brit Isles 1967; 7: 73–75.

[42] Cousens MI. *Blechnum spicant*: habitat and vigor of optimal, marginal, and disjunct populations, and field observations of gametophytes. Bot Gaz. 1981; 142: 251–258.

[43] Schneller JJ. Biosystematic investigations on the Lady Fern (*Athyrium filix-femina*). Plant Syst Evol. 1979; 132: 255–277.

[44] Jacquemyn H, Butaye J, Hermy M. Influence of environmental and spatial variables on regional distribution of forest plant species in a fragmented and changing landscape. Ecography 2003; 26: 768–776.

[45] Fröborg H, Eriksson O. Local colonization and extinction of field layer plants in a deciduous forest and their dependence upon life history features. J Veg Sci 1997; 8: 395–400.

[46] Inghe O, Tamm CO. Survival and flowering of perennial herbs. IV. The behavior of *Hepatica nobilis* and *Sanicula europaea* on permanent plots during 1943–1981. Oikos 1985; 45: 400–420.

[47] Scurfield G. Biological flora of the British Isles. *Deschapsia flexuosa* (L.) Trin. J Ecol. 1954; 12: 225–233.

[48] Hutchinson TC. Biological flora of the British Isles. *Teucrium scorodonia* L. J Ecol. 1968; 56: 901–911.

[49] Peinado Lorca M, Rivas-Martínez S. La vegetación de España. Publicaciones de la Universidad de Alcalá de Henares; 1987.

[50] Costa Tenorio M, Morla C, Sainz H. Los bosques ibéricos Una interpretación geobotánica. Planeta; 2005.

[51] Tyler G. Interacting effects of soil acidity and canopy cover on the species composition of field-layer vegetation in oak/hornbeam forest. Forest Ecol Manag. 1989; 28: 101–114.

[52] Gardner WA. Effect of light on germination of light-sensitive seeds. Bot Gaz. 1921; 71: 249–288.

[53] Heinken T. Dispersal of plants by a dog in a deciduous forest. Bot Jahrb Syst. 2000; 122: 449–467.

[54] von Oheimb G, Schmidt M, Kriebitzsch WU, Ellenberg H. Dispersal of vascular plants by game in northern Germany. Part II: Red deer (Cervus elaphus). Eur J Forest Res. 2005; 124: 55–65.

[55] Brunet J, Valtinat K, Mayr ML, Felton A, Lindbladh M, Bruun HH. Understory succession in post-agricultural oak forests: Habitat fragmentation affects forest specialists and generalists differently. Forest Ecol Manag. 2011; 262: 1863–1871.

[56] Jules ES, Priya S. A broader ecological context to habitat fragmentation: Why matrix habitat is more important that we thought. J Veg Sci. 2003; 14: 459–464.

[57] Fahrig L. Rethinking patch size and isolation effects: the habitat amount hypothesis. J Biogeogr. 2013; 40: 1649–1663.

[58] Tutin TG, Heywood VH, Burguess NA, Moore DM, Valentine SH, Walters SM et al Flora Europaea. 5 vols Cambridge University Press; 1964–1980.

