# Correction: Detecting Fragmentation Extinction Thresholds for Forest Understory Plant Species in Peninsular Spain

**DOI:** 10.1371/journal.pone.0132199

**Published:** 2015-07-01

**Authors:** The PLOS ONE Staff

In the Funding section, the grant number from funder Ministry of Economy and Competitiveness of Spain is listed incorrectly. The correct grant number is: CGL2013-48768-P. The publisher apologizes for the error. The correct funding information is as follows: This study was sponsored by a research grant from the Ministry of Economy and Competitiveness of Spain (CGL2013-48768-P to MÁR). MR was funded by the Spanish Ministry for Education and Science (BVA-2010-0596). IM-C was funded by the Integrated Program of IC&DT (No. 1/SAESCTN/ALENT-07- 0224-FEDER-001755). The funders had no role in study design, data collection and analysis, decision to publish, or preparation of the manuscript.
